# Urinary Neurotransmitter Patterns Are Altered in Canine Epilepsy

**DOI:** 10.3389/fvets.2022.893013

**Published:** 2022-05-16

**Authors:** Teresa Schmidt, Sebastian Meller, Steven R. Talbot, Benjamin A. Berk, Tsz H. Law, Sarah L. Hobbs, Nina Meyerhoff, Rowena M. A. Packer, Holger A. Volk

**Affiliations:** ^1^Department of Small Animal Medicine and Surgery, University of Veterinary Medicine, Hannover, Germany; ^2^Institute for Laboratory Animal Science, Hannover Medical School, Hannover, Germany; ^3^BrainCheck.Pet^®^ – Tierärztliche Praxis für Epilepsie, Sachsenstraße, Mannheim, Germany; ^4^Department of Clinical Science and Services, Royal Veterinary College, Hatfield, United Kingdom

**Keywords:** neurotransmitter, epilepsy, biomarker, urinary, canine

## Abstract

Epilepsy is the most common chronic neurological disease in humans and dogs. Epilepsy is thought to be caused by an imbalance of excitatory and inhibitory neurotransmission. Intact neurotransmitters are transported from the central nervous system to the periphery, from where they are subsequently excreted through the urine. In human medicine, non-invasive urinary neurotransmitter analysis is used to manage psychological diseases, but not as yet for epilepsy. The current study aimed to investigate if urinary neurotransmitter profiles differ between dogs with epilepsy and healthy controls. A total of 223 urine samples were analysed from 63 dogs diagnosed with idiopathic epilepsy and 127 control dogs without epilepsy. The quantification of nine urinary neurotransmitters was performed utilising mass spectrometry technology. A significant difference between urinary neurotransmitter levels (glycine, serotonin, norepinephrine/epinephrine ratio, ɤ-aminobutyric acid/glutamate ratio) of dogs diagnosed with idiopathic epilepsy and the control group was found, when sex and neutering status were accounted for. Furthermore, an influence of antiseizure drug treatment upon the urinary neurotransmitter profile of serotonin and ɤ-aminobutyric acid concentration was revealed. This study demonstrated that the imbalances in the neurotransmitter system that causes epileptic seizures also leads to altered neurotransmitter elimination in the urine of affected dogs. Urinary neurotransmitters have the potential to serve as valuable biomarkers for diagnostics and treatment monitoring in canine epilepsy. However, more research on this topic needs to be undertaken to understand better the association between neurotransmitter deviations in the brain and urine neurotransmitter concentrations in dogs with idiopathic epilepsy.

## Introduction

Dogs and humans are affected by naturally occurring epilepsy, a complex brain disorder characterised by a predisposition to experience recurring seizure events (1–3). It is one of the most common chronic neurological diseases in both species, with many shared clinical and epidemiological characteristics (4–6). Around two-thirds of the affected dogs and half of the human patients do not become seizure free, despite pharmacological treatment (7, 8). Persistent uncontrollable seizures are a health concern increasing mortality, causing psychological and physical stress, and culminating in a negative impact on the overall quality of life (9, 10).

Epileptic seizures are initiated by abnormally excessive or synchronous neuronal activity in the cerebral cortex or hippocampus of the brain (11). The exact pathogenesis of this process has not yet been solved. However, a contributing factor to the underlying pathophysiology of seizures may arise from the imbalance of excitatory and inhibitory neurotransmission, caused by neurotransmitter and receptor alterations (12, 13).

In the past decades, evidence in humans and primates has suggested that seizures were correlated to altered neurotransmitter concentrations of glutamate, ɤ-aminobutyric acid (GABA) and serotonin, which were measured in the extracellular fluid, cerebrospinal fluid (CSF) and serum (14–17). A deviating neurotransmitter composition, caused by a dysfunctional neurotransmitter metabolism in humans, can also result in seizures and other neurological signs (18).

Emerging seizures can also be linked to changes in neurotransmitter receptors. In earlier studies, the altered GABA or dopamine (DA) receptor density was accompanied by seizures or seizure susceptibility in human patients and rodents (19–22). Changes in receptor function, such as binding potential or endogenous activity of glutamate, GABA or serotonin receptors were found in humans suffering from temporal lobe epilepsy (23–27). Additionally, a divergent composition of glutamate or GABA receptor subunits has also been associated with recurring seizures in animal models and humans (28–31). Those detected subunit compositions were similar to those of the more excitatory immature brain and facilitated further seizures and epileptogenesis (32).

In the central nervous system (CNS), glutamate is the major excitatory neurotransmitter, whereas GABA is the primary inhibitory counterpart (33, 34). The equilibrium of these two neurotransmitters maintains the balance of cell excitability. The aforementioned alterations that affect one or both of these neurotransmitters are likely to elicit a shift to arousal in the brain, followed by seizures (13). The neurotransmitter serotonin is known for its anticonvulsant properties and regulation of mood and cognition (35, 36). Therefore, disturbances in the serotonergic system are assumed to evoke seizures and promote frequently developed neurobehavioural/-psychiatric comorbidities associated with epilepsy in dogs and humans (37–43).

In the body, intact neurotransmitters of the CNS are transported through the blood-brain barrier (BBB) to the peripheral systemic circulation, from where they are primarily excreted through the kidney into urine (44–47). The neurobiological basis of this process is poorly understood. It is substrate-specific and can vary for each neurotransmitter (48). However, several animal studies demonstrated an association between central and peripheral neurotransmitter output into the urine (49–52). Moreover, positive correlating neurotransmitter concentrations of serine, glycine and norepinephrine (NE) between the CSF, blood and urine in dogs were recently revealed, emphasising a connection (53).

In human medicine, non-invasive urinary neurotransmitter analysis is used to manage medical conditions such as depression and attention-deficit hyperactivity disorder (ADHD) (54–57). Patients affected by depressive and anxiety symptoms showed increased urinary catecholamines, like NE and epinephrine (E) (58–60). Suicide attempts in depression were strongly associated with urinary excreted DA, even greater than the CSF concentration (61). ADHD symptoms correlated with alterations of the urinary catecholamines NE and E, and a decrease in urinary phenylethylamine (PEA), which is linked to inattentiveness (62–66).

Urinary neurotransmitter analysis is not as yet used for epilepsy management in either humans or dogs, to the authors' knowledge. However, promising evidence was provided in a recent study, indicating altered urinary neurotransmitter patterns associated with the treatment efficacy of medium-chained triglyceride (MCT) oil in drug-resistant canine epilepsy (67). Intake of MCT oil increased urinary GABA concentration in dogs with IE. Also, the GABA/glutamate ratio changed significantly by decreased glutamate levels compared to GABA levels in dogs affected by epilepsy. Furthermore, non-responders without a reduction in seizure frequency below 50% excreted higher glutamate, histamine and serotonin levels in their urine (67).

This study investigated whether urinary neurotransmitter profiles differ between dogs with epilepsy and non-epileptic controls. We hypothesised that urinary neurotransmitter analysis could provide a non-invasive diagnostic tool, where characteristic neurotransmitter deviations can serve as valuable biomarkers in epilepsy research and clinical management.

## Materials and Methods

### Sample Acquisition

In this multicentre study a total of 223 urine samples were collected from 190 privately owned dogs (both sexes; mixed or pure breed) and divided into two cohorts. From the first cohort, 96 urine samples from 63 subjects with idiopathic epilepsy (IE) were obtained. Dogs in the IE cohort had no acute or chronic diseases of the gastrointestinal tract, kidney, liver or heart failure. They met at least the requirements of Tier I (*n* = 15) confidence level of the International Veterinary Epilepsy Task Force (IVETF) for the diagnosis of IE, however, most dogs met Tier II (*n* = 48). Two adjustments to IVETF criterion were applied, as long as magnetic resonance imaging was unremarkable: firstly, abnormalities in the interictal neurological examination caused by antiseizure drug (ASD) treatment were tolerated and secondly, the maximum age at seizure onset was increased to 12 years (68). Samples were collected and analysed as part of three former epilepsy studies, between October 2012 and September 2017, at international study sites: Queen Mother Hospital for Animals, Royal Veterinary College, London, UK (RVC) (*n* = 59: 29 paired samples collected from the same individual at certain study stages; 30 unpaired); University of Veterinary Medicine Hannover, Hannover, Germany (TiHo) (*n* = 3, paired); University of Helsinki, Helsinki, Finland (*n* = 1, paired) (69–72).

The second cohort was a control group of 127 healthy dogs. All control cohort subjects were at least 1 year of age, did not receive any medication and had no chronic diseases. One hundred (*n* = 100) of the second cohort samples were collected from dogs owned by TiHo staff and students, between January and June 2020. The remaining 27 samples were obtained from healthy control dogs at the RVC study site.

To avoid bias of the study results, the dogs were not fed milk products, fruits and vegetables 48 h before sample acquisition. Exposition to strenuous exercise was also avoided for 24 h before sampling. Bitches were not in heat during the collection process. The urine samples were collected via the free catch method. The first or second void of morning urine from the fasting dog (preferable midstream) was used for urinary neurotransmitter analysis. Samples were transferred into a tube containing a preservative to ensure sample stability (50 mg oxalic acid/10 ml urine), followed by an immediate transport to the TiHo laboratory. Samples from the other study sites (London, Helsinki) were collected as part of an enrolment or study visit for epilepsy trials with MCT, epilepsy behaviour studies or from healthy controls of the previously mentioned studies and were directly cooled on ice (69–72).

### Sample Preparation and Analysis

Samples were aliquoted and quickly frozen at the different study site laboratories. They were stored at −80°C for at least 4–6 h prior to shipment. The preserved urine samples were continuously frozen and shipped on dry ice for external analysis of neurotransmitter concentrations to “Doctor's Data,” St. Charles, IL, USA. Nine urinary neurotransmitter levels (serotonin, histamine, glycine, phenylethylamine, DA, E, NE, glutamate, GABA) were quantified utilising High-performance liquid chromatography triple-quadrupole mass spectrometry/mass spectrometry (HPLC-QqQ MS/MS) technology. In addition, creatinine levels were measured by Enzymatic Colorimetric—Kinetic Jaffé method. Those were used as a reference value to determine urine concentrations and to evaluate neurotransmitter levels relative to creatinine levels. The applied neurotransmitter screening method is usually utilised in human patients. In previous canine studies, the method was used multiple times and the archived data revealed biologically reasonable results for this species as well (53, 67).

### Statistical Analysis

Statistical analyses were performed with the R software (v4.0.3) to test the hypothesis, that there is a difference between the urinary neurotransmitter excretion of dogs affected by epilepsy and a healthy control group (H1) (73). Additionally, whether the urinary neurotransmitter excretion of dogs with epilepsy is affected by ASD administration was explored by comparing neurotransmitter levels of ASD- treated and untreated dogs with epilepsy (H2). First, data were log10-transformed to compensate for wide ranges. Then, the transformed data were tested against the hypothesis of normal distribution using Shapiro–Wilk's test. Finally, in the case of normally distributed data, group comparisons were analysed with an unpaired two-sided two-sample *t*-test. When data did not follow a normal distribution, the Wilcoxon-Mann-Whitney test was used in the analysis. Next, multiple group comparisons were analysed with a one-way analysis of variance (ANOVA) to find between-factor differences. Finally, a Games-Howell *post-hoc* test with the Holm correction for multiple comparisons was used to analyse multiple group contrasts and compensate for potential heteroscedasticity. If multiple group data did not follow a normal distribution, the Kruskal-Wallis test was used. Results were considered significant at the following *p*-value thresholds: *p* ≤ 0.05 (^*^), *p* ≤ 0.01 (^**^), *p* ≤ 0.001(^***^), and *p* ≤ 0.0001 (^****^).

## Results

### Study Population

For the current study, 223 urine samples from 190 dogs of more than 21 breeds were collected, including the following: Australian Shepherd (*n* = 5), Beagle (*n* = 6), Belgian Shepherd (*n* =2 ), Bernese Mountain Dog (*n* = 1), Border Collie (*n* = 4), Chihuahua (*n* = 2), Dachshund (*n* = 3), Boxer (*n* = 1), German Shepherd (*n* = 4), French Bulldog (*n* = 1), Golden Retriever (*n* = 2), Havanese (*n* = 1), Jack Russell Terrier (*n* = 1), Labrador (*n* = 2), Vizsla (*n* = 3), Maltese (*n* = 1), Poodle (*n* = 2), Rhodesian Ridgeback (*n* = 1), Siberian Husky (*n* = 2), cross breeds (*n* = 46) and other breeds (*n* = 43). For *n* = 57 dogs, no information of their breed was available. The study population consisted of *n* = 89 males, of which *n* = 32 were intact, *n* = 57 were neutered, and *n* = 98 females, of which *n* = 37 were intact and *n* = 61 were neutered. For three dogs, the gender status is not available. The dogs had a mean age of 5.31 (±SD 3.41) years and weighed a mean of 20.08 (±SD 12.25) kg. Of the *n* = 63 dogs with IE, *n* = 42 were treated with phenobarbital (66.67%) and *n* = 27 were treated with potassium bromide (42.86%), of which 26 received potassium bromide additional to the administered phenobarbital, and one dog was solely treated with potassium bromide. Forty-four (*n* = 44) dogs in the IE cohort received additional ASD treatment in addition to or instead of the aforementioned ASDs (69.84%): levetiracetam (*n* = 16 chronically; *n* = 3 pulse therapy) (74), imepitoin (*n* = 6), Gabapentin (*n* = 2), rectal diazepam rescue therapy (*n* = 7), MCT oil (*n* = 36), cannabidiol oil (*n* = 2), coconut oil (*n* = 1). Seven (*n* = 7) dogs with IE did not receive ASD treatment at the time of sample acquisition and for *n* = 6 dogs no treatment data were available. Thirty-three (*n* = 33) (52.38%) of the affected dogs had at least three generalised seizures in the past 3 months before study enrolment. They were chronically treated with at least one ASD without improving seizure frequency. Seven (*n* = 7) dogs (11.11%) of the IE cohort were seizure free during the past 3 months before sample collection. For *n* = 23 dogs, the seizure frequency was not accessible.

### Neurotransmitter Analysis

**Figure 1:**
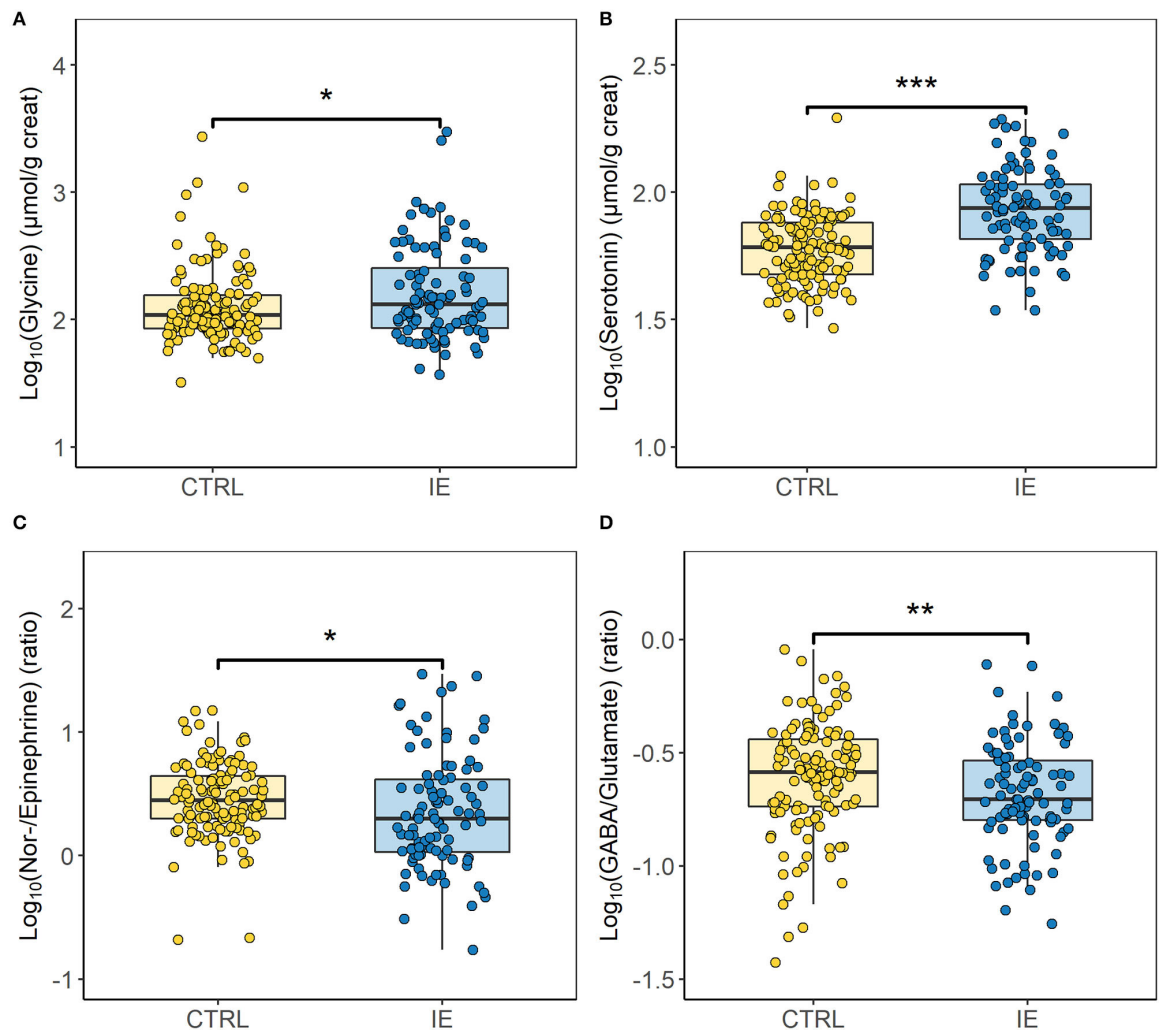
Differences in the urinary neurotransmitter profile between dogs with idiopathic epilepsy (IE) (*n* = 63 dogs/96 samples) and the healthy control group (CTRL) (*n* = 127 dogs/127 samples). Figure shows an increase of urinary **(A)** glycine (*p* ≤ 0.05) and **(B)** serotonin (*p* ≤ 0.001) concentrations and a decrease of the **(C)** norepinephrine/epinephrine ratio (*p* ≤ 0.05) and the **(D)** ɤ-aminobutyric acid (GABA)/glutamate ratio (*p* ≤ 0.01) in dogs affected by idiopathic epilepsy. Data are presented as box-and-whisker plots (the median is represented by the central line, the 25th and the 75th percentile represent the lower and upper limit, and the length of the whiskers represent the 1.5 multiple of the interquartile range). Unpaired two-sided two-sample *t*-test and Wilcoxon–Mann–Whitney test were used to compare the urinary neurotransmitter excretion between dogs with epilepsy and the healthy control group. *p ≤ 0.05, **p ≤ 0.01 and ***p ≤ 0.001.

**Figure 2:**
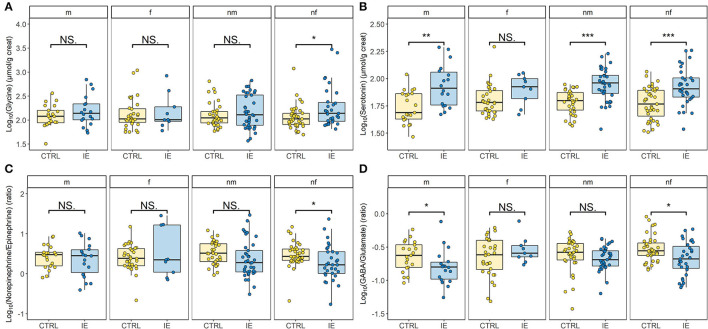
Effects of the sex and neutering status on the urinary neurotransmitter profile of dogs with idiopathic epilepsy (IE) compared to the healthy control group (CTRL). **(A)** Glycine levels differ significantly in neutered females (nf) (*p* ≤ 0.05) (IE: *n* = 22 dogs/31 samples vs. CTRL: *n* = 39 dogs/39 samples). **(B)** Serotonin levels differ significantly in intact males (m) (*p* ≤ 0.01) (IE: *n* = 11 dogs/18 samples vs. CTRL *n* = 21 dogs/21 samples), neutered males (nm) (*p* ≤ 0.001) (IE: *n* = 24 dogs/37 samples vs. CTRL *n* = 33 dogs/33 samples) and neutered females (*p* ≤ 0.001) (IE: *n* = 22 dogs/31 samples vs. CTRL *n* = 39 dogs/39 samples). **(C)** The norepinephrine/epinephrine ratio differs significantly in neutered females (*p* ≤ 0.05) (IE: *n* = 22 dogs/31 samples vs. CTRL *n* = 39 dogs/39 samples). **(D)** The ɤ-aminobutyric acid (GABA)/glutamate ratio differs significantly in intact males (*p* ≤ 0.05) (IE: *n* = 11 dogs/18 samples vs. CTRL *n* = 21 dogs/21 samples) and neutered females (*p* ≤ 0.05) (IE: *n* = 22 dogs/31 samples vs. CTRL *n* = 39 dogs/39 samples). In intact females (f) no significant difference (NS.) of all analysed neurotransmitter between the groups was found (IE: *n* = 5 dogs/9 samples vs. CTRL *n* = 21 dogs/21 samples). Data are presented as box-and-whisker plots (the median is represented by the central line, the 25th and the 75th percentile represent the lower and upper limit, and the length of the whiskers represent the 1.5 multiple of the interquartile range). Unpaired two-sided two-sample *t*-test and Wilcoxon–Mann–Whitney test were used to compare the urinary neurotransmitter excretion between dogs with epilepsy and the healthy control group. *p ≤ 0.05, **p ≤ 0.01 and ***p ≤ 0.001.

**Figure 3:**
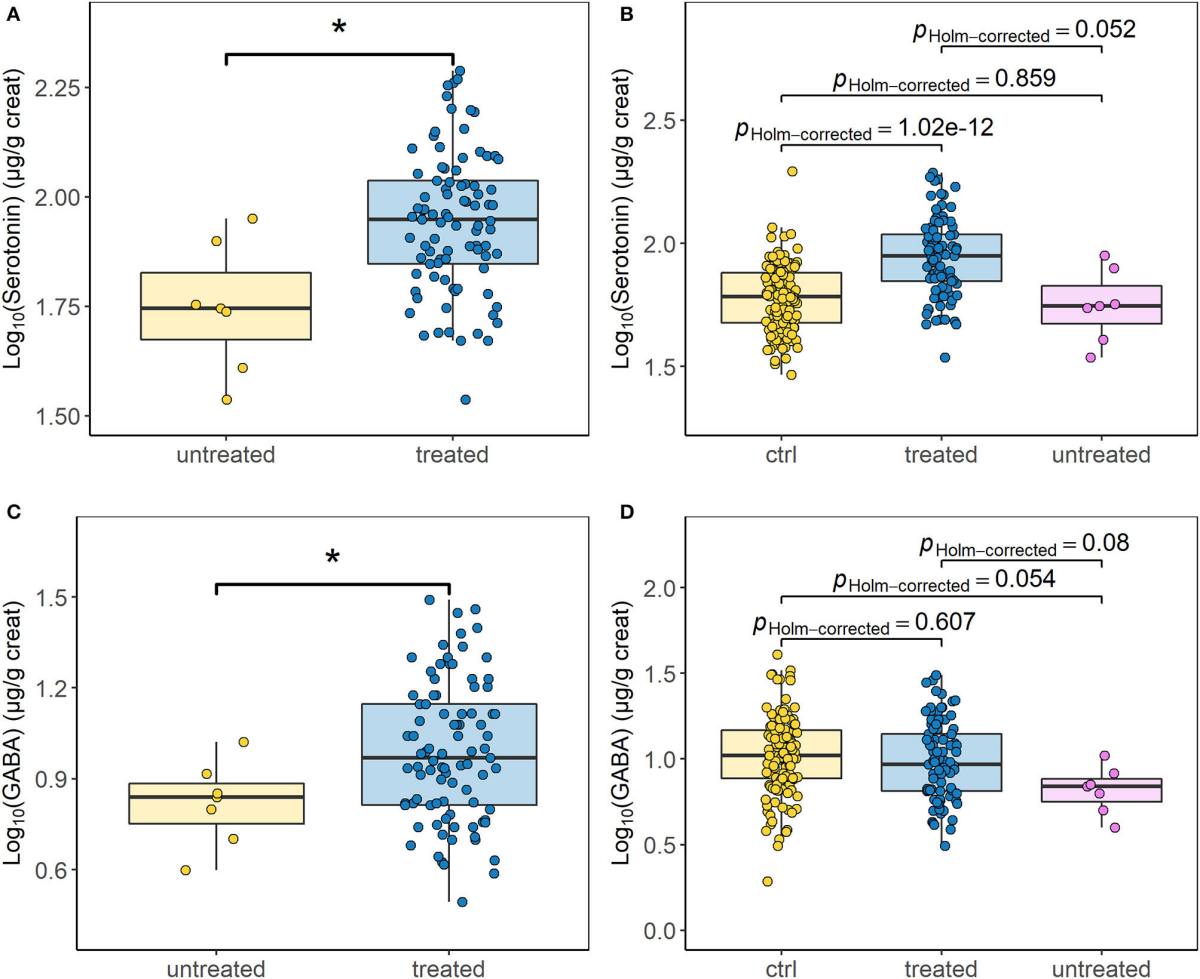
Influence of the antiseizure drug treatment on the urinary neurotransmitter profile. **(A)** Urinary serotonin concentration compared between treated (*n* = 56 dogs/89 samples) and untreated dogs (*n* = 7 dogs/7 samples) affected by idiopathic epilepsy (*p* ≤ 0.05). **(B)** Multiple group comparison of the urinary serotonin excretion between treated (*n* = 56 dogs/89 samples) and untreated dogs (*n* = 7 dogs/7 samples) with idiopathic epilepsy and the healthy control group (ctrl) (*n* = 127 dogs/127 samples). **(C)** Urinary ɤ-aminobutyric acid (GABA) concentration compared between treated (*n* = 56 dogs/89 samples) and untreated dogs (*n* = 7 dogs/7 samples) with idiopathic epilepsy (*p* ≤ 0.05). **(D)** Multiple comparisons of the urinary ɤ-aminobutyric acid excretion between treated (*n* = 56 dogs/89 samples) and untreated dogs (*n* = 7 dogs/7 samples) with idiopathic epilepsy and the healthy control group (*n* = 127 dogs/127 samples). Data are presented as box-and-whisker plots (the median is represented by the central line, the 25th and the 75th percentile represent the lower and upper limit, and the length of the whiskers represent the 1.5 multiple of the interquartile range). Unpaired two-sided two-sample *t*-test and Wilcoxon–Mann–Whitney test were used for group comparison. For multiple group comparisons a one-way analysis of variance (ANOVA) and a Games-Howell *post-hoc* test with the Holm correction was utilised. *p ≤ 0.05.

A significant difference between urinary neurotransmitter levels of dogs diagnosed with IE and the control group was revealed, when sex and neutering status were accounted for (Figures 1, 2). Urinary glycine (*p* ≤ 0.05, Figure 1A) and serotonin concentration (*p* ≤ 0.001, Figure 1B) were significantly increased in dogs with IE. Whereas, the NE/E ratio (*p* ≤ 0.05, Figure 1C) and the GABA/glutamate ratio (*p* ≤ 0.01, Figure 1D) was significantly decreased in dogs with epilepsy. The sex and neutering status of the dogs substantially affected the urinary neurotransmitter excretion (Figure 2). Glycine concentration was significantly increased in neutered females with epilepsy (*p* ≤ 0.05, Figure 2A). The serotonin concentration was significantly increased in intact males (*p* ≤ 0.01), neutered males (*p* ≤ 0.001) and neutered females (*p* ≤ 0.001) with epilepsy (Figure 2B). The NE/E ratio was significantly decreased in neutered females (*p* ≤ 0.05, Figure 2C) with epilepsy. The GABA/glutamate ratio was significantly reduced in intact males (*p* ≤ 0.05) and neutered females (*p* ≤ 0.05) with epilepsy (Figure 2D). Finally, an influence of ASD treatment on urinary neurotransmitter excretion was observed in dogs with epilepsy (Figure 3). Treatment significantly increased serotonin concentration in dogs with epilepsy compared to untreated dogs (*p* ≤ 0.05, Figure 3A), to an even higher level as in healthy controls (p Holm-corrected = 1.02e-12, Figure 3B). GABA concentration was significantly decreased in untreated dogs with epilepsy compared to those treated dogs with IE (*p* ≤ 0.05, Figure 3C). The ASD treatment increased GABA concentration and increased it to a similar level to healthy control dogs (p Holm-corrected = 0.607, Figure 3D). For the remaining urinary neurotransmitters (histamine, PEA, DA, E, NE, glutamate) no statistically significant differences between the two cohorts or an ASD treatment effect were identified (Supplementary Table 1).

## Discussion

The objective of this study was to evaluate the suitability of urinary neurotransmitter analysis as a non-invasive diagnostic tool, where characteristic neurotransmitter deviations serve as valuable biomarkers for canine IE. It was hypothesised that urinary neurotransmitter profiles differ between dogs with epilepsy and healthy controls (H1). Sex and neutering status substantially affected urinary neurotransmitter excretion. In the present study, urinary neurotransmitter patterns were significantly altered in dogs with IE, when sex and neutering status were accounted for, which confirmed the first hypothesis of our study. Urinary glycine and serotonin concentration were significantly increased in dogs with IE, whereas the GABA/glutamate ratio and the NE/E ratio was significantly decreased. Additionally, it was hypothesised that the urinary neurotransmitter excretion of dogs with epilepsy was affected by administered ASD, with hypothesised differences between ASD-treated dogs compared to untreated dogs with epilepsy (H2). Results demonstrated that ASD treatment increased GABA concentration in dogs with epilepsy to the level seen in the healthy control population.

Glycine serves primarily as an inhibitory neurotransmitter in the CNS (75). It generally improves mood, mental performance and memory skills (76, 77). However, elevated levels can compromise cognitive processing and provoke seizures (78, 79). In humans, a rare inherited error of glycine metabolism, called non-ketotic hyperglycinemia, causes an excessive accumulation of this neurotransmitter in the body, particularly in the nervous system (80). Clinical signs of this disease include refractory seizures, hyperactivity and in adults cognitive impairment (79, 80). Affected patients also excrete high levels of glycine in their urine (80). Non-ketotic hyperglycinemia and epilepsy are two different diseases, however, parallels in clinical signs exist. The most prominent clinical sign of human and canine epilepsy are recurrent seizures. Cognitive impairments and hyperactivity are often associated as well (81–87). The elevated urinary glycine levels in dogs with IE found in the current study are another similarity. The results indicating, increased glycine concentration might be a contributing factor inducing seizures and associated cognitive impairment, as well as hyperactivity in affected patients. However, elevated urinary glycine in dogs with epilepsy found in this study, should be differentiated from the massively increased concentrations in human patients with non-ketotic hyperglycinemia. Further studies are needed to evaluate whether urinary glycine can serve as a potential biomarker in canine epilepsy, too. It must be considered, that for laboratory diagnosis of non-ketotic hyperglycinemia CSF and serum glycine concentrations are determined. In a previous study canine glycine levels correlated between CSF, serum and urine, suggesting non-invasive urinary neurotransmitter analysis as a good option for glycine screening in dogs (53). A treatment effect of the ASDs (phenobarbital and potassium bromide), which might have caused the detected glycine increase, was not revealed in this study. For valproate, an anticonvulsive drug administered in human medicine, an elevating effect on urine and plasma glycine levels exists (88). To the authors' knowledge, such an effect is not known from first and second-line drugs (phenobarbital and potassium bromide) authorised for canine epilepsy treatment.

Urinary serotonin levels were increased in dogs with IE, compared to dogs without epilepsy and were substantially affected by their sex and neutering status. These findings match those of a recent study in which urinary serotonin excretion was altered after ovariohysterectomy in bitches (89). Serotonin plays a role in regulating sleep, appetite and mood (36). Grouping the data into treated and untreated epileptic dogs revealed that untreated dogs with epilepsy excreted significantly lower urinary serotonin levels than ASD-treated dogs with epilepsy or healthy control dogs. Decreased serotonin concentrations are related to the pathogenesis of various psychiatric and neurological disorders (41). Alterations in the serotonergic system can lower the seizure threshold and are associated with frequently co-occurring neurobehavioural comorbidities (35, 37). Psychological conditions/behavioural abnormalities are commonly treated with selective serotonin reuptake inhibitors (SSRIs) in humans and dogs (90, 91). However, drug manuals suggest that SSRIs are contraindicated in dogs with epilepsy or a history of seizures (92). In contrast, the International League Against Epilepsy and experimental data suggest SSRIs to be of low risk to patients with a history of seizures or epilepsy, indicating that they can be cautiously used for the treatment of anxiety in some epilepsy patients (93). Some experimental data even exists that SSRIs might be anticonvulsive. For example, the SSRI fluoxetine is effective in dogs with fly catching syndrome, a condition which has been considered by some as limbic epilepsy, but others as a compulsive behavioural disorder (94). In the current study, ASD treatment significantly increased the serotonin concentration in dogs with epilepsy compared to untreated dogs, to an even higher level than in healthy controls dogs. Elevated serotonin levels can be an amplifying and beneficial effect of ASD treatment, due to the protective properties of serotonin against seizures. Increased serotonin concentration may also improve associated neurobehavioural disorders in affected dogs, without prescribing contraindicated SSRIs, however, further studies are required to explore this potential positive effect.

The current study also revealed a diminished GABA/glutamate ratio in dogs with IE, which reflects low GABA levels or high glutamate levels in the examined urine samples, respectively. Both neurotransmitters are amino acids with contradictory effects on the body. GABA acts as the primary inhibitory neurotransmitter, while glutamate is the major excitatory counterpart in the CNS (33, 34). These findings in the urine potentially mirror neurotransmitter alterations in the epileptic brain. Furthermore, low urinary GABA concentrations in drug naïve dogs, compared to treated dogs with IE and healthy controls were shown. Dogs with IE who received ASD treatment excreted a higher urinary GABA concentration, which was almost at the same level as healthy controls. These findings reflect the expected lower GABA concentration in untreated epilepsy and corroborate a treatment effect, which may have corrected the GABA values up to the healthy controls state. However, the ASD administered in this study (phenobarbital and potassium bromide) are not known to directly influence GABA concentrations. Their anticonvulsant effect is mediated by other action mechanisms, including GABA receptor interactions (95, 96). Ultimately, however, acute and chronic phenobarbital treatment reduce brain GABA levels (97). Why urinary GABA levels behave differently requires further research.

The NE/E ratio was found to be decreased in dogs affected by IE, representing low NE levels or high E levels in the examined urine samples, respectively. These monoamine neurotransmitters are catecholamines and act receptor-binding-dependent either as excitatory or inhibitory stimulants in the CNS (98, 99). NE is known for its anticonvulsant properties in epilepsy, even though it can also be proconvulsive under certain circumstances (13, 100–102). Reduced NE levels of dogs with IE compared to healthy controls in the presented study corroborate the generally anticonvulsive effect of this neurotransmitter. The lack of NE might contribute to epileptogenesis and induction of seizures in the examined dogs. Furthermore, NE affects cognition, attention and memory ability (103). The noradrenergic system changes cause various neuropsychiatric and -degenerative disorders, such as Alzheimer's disease and ADHD in humans (103–105). As aforementioned, canine epilepsy can be associated with cognitive impairments (72, 82–84). The low NE concentrations detected in this study might contribute to the development of those impairments. These findings consistent with those of decreased NE levels assessed in the brain of human patients with Alzheimer's disease, which were correlated with the degree of cognitive impairment (106). Another comorbidity of human epilepsy is ADHD, with behavioural similarities also documented in canine epilepsy patients (86, 87). Previous research has indicated that ADHD can be associated with imbalances in the noradrenergic and dopaminergic systems (105, 107). Several studies reported a correlation between ADHD in children and altered urinary catecholamine excretion (56, 63, 108). Pliszka et al. detected elevated urinary excretion of NE metabolites in children with ADHD compared to healthy controls and increased urinary E excretion when ADHD was accompanied by anxiety (62). Anxiety disorders are also common in human and canine epilepsy (39, 109). A relationship between increased anxiety and exaggerated stress response of the neuroendocrine system haven been previously described (60, 110). E regulates many important body functions and is substantially involved in stress response (99). Elevated E concentrations in dogs with IE may have caused the identified deviation in the urinary NE/E ratio and may also be responsible for the co-occurring anxiety in canine epilepsy. Finally, sleep disturbances are often associated with epilepsy in humans (111). They are assumed to occur in dogs as well, although evaluation remains difficult (112). In former studies, poor sleep quality and disordered sleep were linked to a profuse activation of the sympathetic nervous system, resulting in increased nocturnal serum catecholamine levels (113, 114). As a comorbidity of epilepsy, altered sleeping patterns might also have existed in the canine participants of this study. This may have caused elevated nocturnal E levels, which were excreted and detected in the analysed morning urine. Overall, the evidence presented indicates that alterations in the NE/E ratio of dogs with IE in the current study may be associated with the development of seizures and common comorbidities, such as sleep disturbances, ADHD- and anxiety-like behaviour. The results of this study suggest a potential role of the nor-/adrenergic pathway alterations in canine epilepsy and neurobehavioural comorbidities.

A few limitations of the present study should be noted. First, the multicentred sample acquisition enabled a large sample size of urine from participants with IE and healthy controls, however, variability in sample collection and storage may have impacted results. The number of untreated dogs with IE concerning the total study population of dogs with epilepsy was small, therefore, caution must be applied, as findings referring to this population might not be representative. Another limitation of this study is that a direct correlation between CNS and urinary neurotransmitters levels has only been shown to a limited extent in previous research. Finally, despite us finding differences, these might not be clinically discriminatory and only be considered as a monitoring tool. Future studies are needed to identify for which patient these changes are clinically relevant.

Numerous factors can influence the eliminated urinary neurotransmitter concentrations. Neurotransmitter passage from the CNS to the periphery is regulated by the BBB, being formed by specific endothelial cells, through which the transport differs for each substrate (48). For glycine a non-carrier-mediated process for BBB crossing is assumed in rats, whereas for dogs no significant transfer through the BBB could be shown so far (115, 116). Serotonin is shuttled via a serotonin transporter, which enables a bidirectional permeation through the luminal membrane of the endothelial cells, but only unidirectional transport to the brain on the abluminal side (117, 118). GABA can cross the abluminal endothelial membrane through a transport system and a luminal membrane passage is presumed as well, even though the transporter has not yet been identified (45, 119). Glutamate can also pass the BBB across the abluminal side via several transporters into the endothelial cells, from where a bidirectional luminal transport is possible (44, 120). NE is shuttled via an abluminal transporter out of the brain into the endothelial cells (117, 121). E is proven to be assimilated into endothelial cells, however the exact process remains elusive (122). BBB function might be altered during seizure and the neurotransmitter could pass more readily. The presented evidence emphasises that the neurotransmitter transfer through the BBB is still not completely revealed. In the interpretation of the current study results, substrate-specific permeability and transport directions of the respective neurotransmitters through the BBB endothelial cells, should be considered.

After crossing the BBB, the neurotransmitters circulate in the bloodstream, from which they are subsequently eliminated by the kidneys into the urine (48). Renal excretion of monoamine neurotransmitters is affected by two mechanisms: glomerular ultrafiltration from the arterial blood and active reabsorption and secretion through specific transporters (48, 52, 123, 124). All participating dogs had normal renal function and were not affected by renal diseases. Nevertheless, the above-mentioned processes can modulate the detected amount of urinary excreted neurotransmitters in healthy dogs as well and this might have affected the acquired results of this study.

Another impact on urinary neurotransmitter levels could arise from additional synthesis outside the CNS. Neurotransmitters are also produced in the peripheral nervous system (PNS), as in serotonin secreting enterochromaffin cells of the enteric nervous system or in norepinephrine producing renal nerves (125–127). Even bacteria, hosted in the body as microbiota, are capable of synthesising neuroactive molecules by themselves (e.g., GABA), or regulating their host's neurotransmitter metabolism (e.g., serotonin), resulting in a modified overall neurotransmitter pool (128–131). Moreover, neurotransmitters are additively produced in many other body organs beside the nervous system, such as the pancreas (e.g., GABA), the adrenal glands (e.g., NE, E, DA) and the kidneys (e.g., GABA, E, NE) (132–137). Ingesting nutritional sources of neurotransmitters or their precursors, can have an ancillary influence on the neurotransmitter pool of the body (128, 138, 139). To minimise this external impact, dairy products, fruits and vegetables were not fed before sample acquisition. However, the dog's personal standard diet, containing meat and seafood as neurotransmitter sources, might also have influenced their urinary neurotransmitter concentration on an individual level (128). It is assumed, that animals and processed foods contain more stable levels of neurotransmitters, than the avoided plants, which might have reduced the individual variety (128). Moreover, endogenous mechanisms of the body, as inactivating enzymes, intestinal metabolism and certain barriers, limiting the effect of nutritional neurotransmitter and as well their urinary excretion (128). Anyhow, nutrition is an important factor for the neurotransmitter metabolism of the body. In further studies it needs to be addresses to which extent dietary factors influence the canine urinary neurotransmitter excretion.

Although the mentioned factors influence on the urinary neurotransmitter concentration, former studies revealed an association between central and peripheral neurotransmitter excretion into the urine. In early studies labelled NE was injected into the cisterna magna of dogs, followed by detection in their blood and fast metabolite excretion via the urine (140). Following these findings, a more recent study showed positively correlating neurotransmitter concentrations of serine, glycine and NE mirrored in three canine body fluids: CSF, blood and urine (53). Orally administered serotonin substrates in rats enhanced the serotonergic activity in the CNS and urinary serotonin levels, indicating a shared regulation mechanism (49). In another study injecting a neurotoxic compound into rat brains induced diminished DA levels in their brain and urine (50). Furthermore, a relationship between urinary excreted neurotransmitters and psychological disorders in humans has been identified. Elevated concentrations of urinary catecholamines, such as NE and E were associated with depression and anxiety (58–60). Urinary excreted DA correlated with suicide attempts in depressed patients even stronger than the CSF levels (61). In addition to the presented evidence, a crosstalk between the CNS and PNS was demonstrated in different studies, further strengthening the central and peripheral neurotransmitter association (141–143).

In human medicine rare inherited disorders exist, which are causing seizures and are also associated with neurotransmitter alterations in the body (144). Disorders of the pyridoxine metabolism evoke ASD resistant seizures in neonates, responding to administered pyridoxine (pyridoxine dependency) or in rare cases solely to its active form, pyridoxal phosphate (pyridoxal phosphate dependency) (144–146). Pyridoxal phosphate is involved in neurotransmitters metabolism of glutamate, GABA and glycine, but the contribution to the epilepsy remains controversial (146–148). Cerebral folate deficiency manifests with late infantile onset seizures and is treatable with folate acid supplementation (149). It is caused by folate transport or metabolism disorders, resulting in low CNS folate concentrations, which can be accompanied with a peripheral folate deficiency (149, 150). Folate is required in the neurotransmitter metabolism of glycine and its influence on serotonin and catecholamine homeostasis is discussed (144, 151, 152). Further inherited neurotransmitter disorders associated with seizures are succinic semialdehyde dehydrogenase (SSADH) deficiency (GABA metabolism disorder), aromatic L-amino acid decarboxylase (AADC) deficiency (dopamine/serotonin synthesising enzyme disorder, seizures are described but uncommon) and the aforementioned non-ketotic hyperglycinemia (glycine metabolism disorder) (80, 153–156). These disorders are not known in dogs so far. However, in canine IE the underlying cause of seizures remains unknown, which might generate a heterogenous group with different yet undiscovered diseases (1). The dogs of the current study met all requirements of the IVETF for the diagnosis of IE (Tier I/Tier II), but the mentioned human disorders require specific diagnostic screening and are not included in these clinical work-up guidelines (68). It is possible that similar inherited metabolic disorders exist in canine patients undetected, evoking seizures and altered neurotransmitter concentration. Nevertheless, in the mentioned human diseases additional abnormalities in the neurological examination/brain imaging are present, which are exclusion criteria of canine IE (Tier I/Tier II) and are therefore considered unlikely (144, 146, 150, 155). The exact underlying pathophysiology of seizures, and the associated urinary neurotransmitter alterations revealed in this study, remains to be elucidated further in the future. The current study can only be seen as a starting point.

Imbalances in the neurotransmitter system that cause epileptic seizures also lead to altered neurotransmitter elimination in the urine of affected dogs and, therefore, can serve as valuable biomarkers in epilepsy. Urinary neurotransmitter analysis with its non-invasive collection technique offers a major advantage over determining neurotransmitters from other body fluids (e.g., CSF, serum). Recent evidence revealed an association between urinary neurotransmitter patterns and treatment efficacy in drug-resistant dogs with IE, suggesting a benefit of utilising this diagnostic tool, particularly in epilepsy patients (67). In the future, neurotransmitter analysis could allow for a better understanding of the underlying pathomechanisms of epilepsy. These biomarkers may indicate specific subtypes of epilepsy in this heterogeneous disease, associated with pharmacoresistance. Applied in a clinical setting, the non-invasive urinary neurotransmitter analysis could be used for individual treatment monitoring and customised adjustments of therapeutic interventions in canine or even human epilepsy.

## Conferences

Preliminary results of the current study were presented at following conferences: 33rd ESVN -ECVN Symposium 2021; 30. Jahrestagung der Fachgruppe “Innere Medizin und klinische Labordiagnostik” der DVG (InnLab) 2022 (awarded with the 1st poster prize).

## Data Availability Statement

The datasets used and/or analysed within the current study are available as Supplementary Material, further inquiries can be directed to the corresponding author.

## Ethics Statement

Written informed consent was obtained from all owners. The study was conducted following the guidelines of the University of Veterinary Medicine Hannover and approved by the thesis committee of the University. In addition, data and urine samples from multiple epilepsy studies were used. These studies were approved by the local Ethics and Welfare Group (EWG) (URN 2011 1132, URN 2016 1558 and URN 2017 1743-2).

## Author Contributions

TS participated in the planning of the study, carried out the main practical work, the recruitment and the sample acquisition of the control group samples in Hannover, interpreted the results, and drafted the manuscript. BB, TL, SH, RP, and HV provided clinical and laboratory data of the dogs with idiopathic epilepsy of former epilepsy trials and studies. HV designed and coordinated the study. SM supported sample acquisition. SM and HV made essential contributions to the conception and acquisition of data. ST performed the statistical analysis and wrote sections of the manuscript. ST, BB, SH, NM, RP, and HV critically reviewed and edited the manuscript for important intellectual content. All authors contributed to the manuscript revision, read and approved the final manuscript.

## Funding

This work was financially supported by the Biotechnology and Biological Sciences Research Council (BBSRC, grant code BB/P001874/1). This Open Access publication was funded by the Deutsche Forschungsgemeinschaft (DFG, German Research Foundation) within the programme LE 824/10-1 “Open Access Publication Costs” and University of Veterinary Medicine Hannover, Foundation.

## Conflict of Interest

BB was employed by company BrainCheck.Pet^®^. The remaining authors declare that the research was conducted in the absence of any commercial or financial relationships that could be construed as a potential conflict of interest.

## Publisher's Note

All claims expressed in this article are solely those of the authors and do not necessarily represent those of their affiliated organizations, or those of the publisher, the editors and the reviewers. Any product that may be evaluated in this article, or claim that may be made by its manufacturer, is not guaranteed or endorsed by the publisher.

## Abbreviations

GABA: ɤ-aminobutyric acid
CSF: cerebrospinal fluid
DA: dopamine
CNS: central nervous system
BBB: blood-brain barrier
NE: norepinephrine
ADHD: attention-deficit hyperactivity disorder
E: epinephrine
PEA: phenylethylamine
MCT: medium-chained triglyceride
IE: idiopathic epilepsy
IVETF: International Veterinary Epilepsy Task Force
ASD: antiseizure drug
RVC: Royal Veterinary College
TiHo: University of Veterinary Medicine Hannover
HPLC-QqQ MS/MS: High-performance liquid chromatography triple-quadrupole mass spectrometry/mass spectrometry
ANOVA: analysis of variance
SSRIs: selective serotonin reuptake inhibitors
peripheral nervous system
SSADH, succinic semialdehyde dehydrogenase, AADC: aromatic L-amino acid decarboxylase.
